# The Cyclopædia of Practical Surgery, &c.

**Published:** 1840

**Authors:** 


The Cyclopædia of Practical Surgery, embracing a complete View of all the Departments in Operative Medicine. Edited by *William B. Costello*, M.D. Member of several Learned Societies, National and Foreign. Part V. London, Sherwood and Co.

The Part before us opens with the conclusion of Mr. *Bennett Lucas's* article on Asphyxia. It displays both information and judgment, and it would be well were the injunctions contained in it carefully studied and acted on by the younger members of the profession.

Mr. Lucas dwells very forcibly and very properly on the dangers of insufflation, practised as rudely as it often, we might almost say generally, is.

Mr. Lucas joins in the now general condemnation of tracheotomy. He gives a brief account of the ingenious instrument of that most ingenious man, Mr. Read. It consists of a syringe or pump which contains by exact measurement fourteen cubic inches; a mouth-piece or guard; a flexible tube for removing the deteriorated air; a coil of metal tube, by which it is proposed to heat the atmospheric air previously to its introduction into the lungs; an Indian rubber bag which is air-proof, and is furnished with a stop-cock. With this apparatus a portion of the deteriorated air may be removed from the lungs in the following manner. The pipe of the mouth-guard is to be placed upon the tongue of the patient, and the guard pressed close to the lips by an assistant; one end of the tube is to be inserted into the mouth-guard, and the other into the pump: the nostrils are then to be closed by an assistant to prevent the admission of air, and by cautiously working the pump not only deteriorated air, but also mucus or any other fluid, can be removed from the windpipe. After each stroke of the pump if the fingers be removed from the nostrils, the pure atmospheric air will instantly rush in.

Mr. Lucas is of opinion that the operation of the instrument will be most materially assisted by the application of Leroy's bandage. He would not, indeed, employ it without conjoining with it alternate pressure and relaxation on the parietes of the chest.

Friction, volatile stimuli to the nostrils, stimulating injections into the stomach, electricity, are all proper subsidiary measures. On electricity Mr. Lucas remarks:—A new method has been proposed by Leroy: he introduced acupuncture needles, one on either side, between the eighth and ninth ribs until they penetrated a short distance into the fibres of the diaphragm; he then completed the galvanic circle by means of a pile of twenty-five or thirty pairs of plates, one inch in diameter, when the diaphragm immediately contracted and depressed the abdominal viscera, and an inspiration was made. The circle was then interrupted, when the diaphragm, urged by the weight of the abdominal viscera, and assisted by a gentle pressure made on the abdomen by the hand, returned to its former position, and an expiration was accomplished. By alternately repeating these operations respiration was re-induced; but when a continuous current was applied, the respiratory movements were irregular. In the experiments of Leroy, which were witnessed by Majendie, animals were often resuscitated after five minutes' submersion by the application of galvanism in the manner just described. Electricity is hardly accessible in private practice, but at the stations for the reception of asphyxiated persons they should be kept and used in the most efficient manner.

Mr. L. states, in reference to the period of submersion in water, compatible with recovery, that it is difficult to lay down any rule by which it can be determined *à priori*, whether our attempts at restoration will be successful, beyond the signs which characterize the existence of total death. Whilst on the one hand, a few minutes' submersion has been sufficient to baffle the most energetic and well-directed measures of the practitioner in his attempts at re-animation, cases on the other hand are on record, where recovery has crowned his efforts after the body has been fifteen, twenty, and even thirty minutes in the water. The longest period recorded by the Humane Society, of recovery after submersion, is three-quarters of an hour.

After noticing in an interesting manner asphyxia from hanging, and observing that he found, after macerating the bones of a criminal that the dentata was fractured at the junction of its laminæ with its body, but, from the little displacement of the fractured pieces, no pressure could have been exerted upon the spinal chord; Mr. Lucas passes to asphyxia from the inhalation of carbonic acid gas. We need not follow him through his account of this, but merely state that Mr. Snow informed him, that he had inspired some pure carbonic acid gas, and that it only produced a tickling of the fauces.

Mr. Lucas thinks very favourably of the use of Mr. Read's apparatus in a case of this kind, its capability of exhausting the lungs appearing demonstrated. Its application, therefore, in those cases of suspended animation, where a gas of the specific gravity of carbonic acid, is in large quantities in the lungs, cannot but be desirable for the purpose of removing a portion of this deleterious agent before sending into these organs pure atmospheric air, or even oxygen itself.

AUSCULTATION is treated shortly and succinctly by Dr. *Benson*. He notices its various applications—among others, that to the detection of calculus in the urinary bladder. The stethoscope may be applied to the pubes or sacrum. The loud clear sound, he says, of calculus, the dull sound of a fungus, and the sort of pumping noise occasioned by moving the sound about in an empty bladder, may be easily distinguished, one from the other, by mediate auscultation, though these different conditions often embarrass the surgeon who trusts to the touch alone.

Laennec had previously directed attention to auscultation of the tympanum, and as the subject of aural diseases is now attracting much attention, we are induced to resuscitate his observations. "If," writes Laennec, "we apply the stethoscope upon the mastoid process of the temporal bone, while the patient inspires forcibly with the nostril of the same side (the other being closed with the finger) we perceive a blowing sound, indicating the penetration of air into the mastoid cells. If there is any moisture in the Eustachian tube, or tympanum, we perceive a gurgling very like that of the mucous rattle; and if the mucus happens to obstruct the tube all sound ceases. From this and other analogous facts we may ascertain the patency, or obliteration, of the Eustachian tube, and may thus be enabled to determine more particularly the cases in which it is proper to attempt curing deafness by throwing injections into this, or by perforating the membrane of the tympanum."

AVULSION, meaning the forcible separation from each other of parts of the body which were previously, more or less, intimately united, is well handled by Dr. *Macfarlane*. He considers it under the heads of *accidental avulsion*, and *avulsion* employed as a *surgical* process.

He alludes, of course, to the circumstance which has long attracted attention—the little tendency to hæmorrhage displayed by lacerated arteries. He observes that the formation of the clot, necessarily occupying a certain time, can furnish no explanation—nor can the retraction of the artery exert much influence, because this is not nearly so complete as when the vessel is simply cut across. He thinks, and so do we, that the explanation of Béclard is the most rational—namely that the obstruction to the hæmorrhage consists in the lacerated inequalities of the inner coat of the artery. Dr. Macfarlane has been enabled to verify this opinion by accurate examination, in two instances. One of the cases was admitted into the Glasgow Royal Infirmary; and the other occurred in private practice. In both, an arm was torn off by machinery. One lived twelve, and the other thirty hours. The inequality of the inner coat of the brachial artery from laceration extended up along the vessel about an inch and a half from its torn extremity. It was, in several places partially detached from the middle coat, so as to form several small projections, which almost completely filled the calibre of the vessel, and must have afforded not a single, but a series of obstructions, to the effusion of blood. In both cases the artery projected about an inch and a quarter beyond the muscles; its extremity was ragged, and its cavity apparently obliterated, so that not a drop of blood escaped. No pulsation could be felt in the pendulous portion, but immediately above it was distinctly recognized. On dividing the artery close to the face of the stump, a jet of blood, corresponding to the size of the vessel, was thrown out, showing distinctly that the previous absence of hæmorrhage was to be attributed to the lacerated inner coat, and annular contraction of the artery.

Dr. Macfarlane points out the usual occurrence of severe shock to the nervous system, and its occasional absence. Sometimes there is wonderfully little pain.

"In the well-known case of Wood the miller, as detailed by Cheselden in the Philosophical Transactions, the arm was torn off, at the shoulder, with such velocity, that the patient was not aware of the accident till he saw his arm moving round on the wheel. A boy in a cotton-factory had his fingers entangled in the machinery, which was in rapid motion. The occurrence was immediately observed by an adult workman, who rushed to his assistance, but so incautiously, as to become himself the chief sufferer. The boy escaped with the loss of his fingers, and the man had his hand torn off above the wrist. He was aware of being injured, but did not know for some time that the hand which he saw hanging on the machinery was his own. A wealthy merchant of Glasgow was riding quickly on horseback, and the night being dark, he came suddenly upon a horse and cart on the road. He threw out his hand against the opposing horse to push it from him, and was not aware till he had gone to a considerable distance, that his thumb was torn off." 449.

Dr. Macfarlane notices the supervention in some instances of tetanus, in other instances, the occurrence of retention of urine. He notices, too, another consequence, by no means unimportant. The torn extremities of the nerves sometimes become the seat and source of excruciating and intractable pains. "This symptom," he adds, "we have uniformly observed after avulsion of the scalp, an accident occurring to women, and that not unfrequently, in our public works. There is at present in the infirmary a young woman who had about two-thirds of the scalp torn off, and the pericranium laid bare, her hair having been caught by machinery. The case is doing well; but she is subject, and has been so since the third or fourth day after the injury, to excruciating neuralgic pains of the head and face, especially on touching slightly the remaining portion of the scalp on the forehead and temples. Occasionally, these pains entirely disappear after the wound has cicatrized, in other cases, however, they continue ever after, and are a source of great suffering."

*Surgical Avulsion* he divides into *simple*, where it constitutes the whole procedure, as in the case of removal of a polypus—or *compound*, where it forms part of another operation. In the latter case it is had recourse to for the removal of deep or vascular parts, which might bleed too freely under excision. Dr. M. remarks, that it can only be safe to adopt this in surgical operations, when the vessels connected with the part to be removed are not of large size, or when they cannot be readily secured by a ligature, as in the extirpation of tumors from the axilla, or behind and below the angle of the jaw. It must be acknowledged, he subjoins, that hæmorrhage rarely happens, either as an immediate, or remote consequence, of the forcible extraction of deep-seated tumors, provided the torn vessels are surrounded by, and enabled deeply to bury themselves in, loose cellular texture. But it is otherwise, when the tumor is situated near a bone, and is firmly adherent to muscular or fibrous texture, no layer of cellular tissue intervening. The force required for its removal from such a situation is not only greater, but the chances of hæmorrhage occurring are so increased, as to call for either the application of ligatures to the lacerated arteries, or the use of firm and steady pressure.

BALANITIS, or inflammation of the mucous membrane of the glans penis, and inner layer of the prepuce is a short article by Mr. *Henry James Johnson.*

After noticing the delicacy of the epithelium of the part, and observing that the extent of the prepuce varies greatly in different persons—in some it is so short that the glans is quite uncovered, in others the glans is partially covered, and in others, again, the prepuce is so long as to conceal the glans, or even to constitute "phymosis"—he goes on to say, that the longer the prepuce, cæteris paribus, the more irritable the mucous membrane, and the greater the liability to collection of the secretion of the sebaceous follicles.

The mucous membrane of the glans and prepuce is liable to inflammation, which may occupy a part or all of it, and may be acute or chronic.

*Acute Balanitis.* This generally comes on suddenly. There is a sense of itching, sometimes of smarting, rarely of sharp pain, within the foreskin, and particularly around the corona glandis. The prepuce is a little swollen, perhaps slightly reddened. From within it, there issues a puriform discharge, usually of dirty colour, often profuse, and sometimes offensive to the smell. It is generally thickened with sebaceous matter; occasionally it is thinner, and more like ordinary pus.

If phymosis exists, the discharge may be supposed to come from the urethra. Its orifice may pout, and the point of the glans may look red and tumid; but, on wiping it with lint, and making pressure along the course of the urethra, no matter is observed to issue from the latter, whilst similar pressure behind the corona, and on the glans itself, occasions its exit from the præputial orifice.

"It may be difficult," says Mr. Johnson, "to determine whether the discharge proceeds from the surface of the mucous membrane, or from ulcerations behind the corona, or on the inner prepuce. But if ulcers exist, there is more tenderness on pressure; and when they have lasted for any length of time, hardness may commonly be felt. This latter symptom is not so characteristic as might, perhaps, be supposed. Warts behind the corona may co-exist with balanitis, and the sensation communicated to the finger, on making pressure from without, is so like what could result from indurated sores, that I have been more than once deceived by it." 454.

If phymosis is not present, the diagnosis is easy, retraction of the prepuce exposing its inner layer and the glans, the seat of the disease. Partial abrasion of the mucous membrane, occasioning a remarkably patchy appearance, is frequently observed.

Balanitis may be attended with inflammatory complications, such as inflammation of the absorbents of the penis, inflammation and enlargement of the lymphatic glands, on the pubes, or in the groins; suppuration in the cellular membrane of the penis is occasionally, though rarely observed. The acute often ends in the chronic form—or (an extreme case) in extensive warts, ulceration of the glans or of the prepuce, and even protrusion of the former through a morbid opening in the latter.

*Causes of Balanitis.*—The great predisposing cause is too long a prepuce, with acrid, if not retained, secretions. The act of connexion is usually the immediate cause. If the venereal orgasm is frequently repeated, or if the secretions of the female are morbid, the occurrence of the disorder becomes the more probable. Severe exertions, a long walk, local friction, however occasioned, all tend to produce it. Free living assists their operation. Substances which disagree with the stomach, and are apt to bring on urticaria or erythematous eruptions on the skin, may excite it. Mr. Johnsnn has seen it occasioned by copaiba. A debauch in some will give rise to it.

*Treatment.*—" If the prepuce admits of retraction, the whole mucous surface should be gently, but effectually, washed with warm water, so as to remove the secretion that adheres to it. Lint dipped in a solution of the liquor plumbi acetatis, of the strength of about two drachms to the pint of water, should then be applied, and retained. The lint should be changed twice or thrice daily, and the tepid ablution employed night and morning. Active aperients should be prescribed, and a cooling regimen, with quiet, should be recommended.

If there is phymosis, and the glans cannot be uncovered, warm water should be injected between it and the prepuce, so as to syringe out, as effectually as possible, the sebaceous matter. This should be followed by similar injections of the diluted liquor plumbi, and the latter ought to be repeated several times in the day."

For the inflammatory complications, leeches, evaporating lotions, antiphlogistic means, in fact, are requisite. If an abscess forms, it must be opened. If it occurs just behind the corona, which it sometimes does, it is very small, often not much larger than a pea, rarely equal in size to a marble. A not unfrequent situation is contiguous to the frænum. It may fairly be presumed that these small abscesses are immediately connected with the sebaceous glands. When opened, either by nature or the lancet, the aperture is apt to continue fistulous. A fine heated wire, introduced once or twice, readily effects a cure.

*Chronic Balanitis.*—Acute balanitis can scarcely happen, unless the investment of the glans by the prepuce is considerable; but the chronic form is not uncommon when the foreskin does not cover more than a third of the glans. The complaint is usually limited to the inner layer of the prepuce contiguous to the corona, and the gutter immediately behind the latter.

This portion of the mucous membrane is red, tumefied, and moist. If there is much prepuce, the sebaceous secretion may collect; if there is little, with ordinary cleanliness, it will not. The surface is not usually abraded of epithelium; but after coition, or much exercise, or a debauch, it may become so. The tendency to "excoriations" in connexion, is marked; and this very tendency predisposes the individual to venereal sores. The subject of chronic balanitis can scarcely indulge in sexual intercourse, without either an abrasion, or ulceration, or some other consequence of the extreme irritability of the part.

The affected surface is generally tender to the touch, particularly after the excitement of coition; the small abscess behind the corona is common.

" When," says Mr. Johnson," the disorder has lasted long, the mucous membrane behind the corona is thickened, warts are apt to spring up, the epithelium is easily abraded, and small ulcers, with a yellow surface, and slightly cupped, are occasionally observed, independently of previous connexion. These ulcers appear to be similar to the sores that are not unfrequent on the mucous membranes of the lips and cheeks, and tongue, in persons with deranged digestive organs. They wear a similar aspect, looking as if a piece of mucous membrane had been picked out with the nail; and they are observed in similar states of constitution. However this may be, such sores on the prepuce do certainly arise spontaneously, and heal without entailing secondary symptoms. I have seen them mistaken for 'chancres,' and patients put unnecessarily on a course of mercury." 455.

Mr. J. thinks that persons with chronic balanitis are peculiarly prone to herpes of the prepuce.

*Causes.*—The essential one is too long a prepuce, the irritability of the mucous membrane occasioned by that being lit up into disease by excesses, dietetic or sexual.

*Treatment.*—This may be palliative or radical. The former consists in ablution with cold water every night and morning, the retention of lint behind the corona, dipped in the diluted liquor plumbi acetatis, or in a solution of the sulphate or acetate of zinc, an occasional application of the lunar caustic, in substance or solution, regulation of the secretions, and avoidance of excesses. If the cuticle is abraded, the black wash, or plain lime water, or a weak solution of the sulphate of zinc, will remedy it. The ulcers alluded to already should be lightly touched every second day with caustic, or dressed with the black-wash.

But these means are far from effectual. The least excess resuscitates the disorder. The only radical remedy is to be found in circumcision, or division of the prepuce. The operation is very successful. Circumcision is the most effectual, but it is the most severe. Division of the prepuce answers well enough.

BANDAGE is, we need not say, well done by Mr. *Chapman*. His account of the different forms of it is clear, and the illustrations are equally so. It is impossible, of course, to give any account of an article of this nature, useful as it is to the younger surgeon.

BLISTER is treated by Mr. *Alexander Ure*, a gentleman of great promise. We shall extract one or two passages from his paper.

The blistering property of the *meloë vesicatorius*, from which our common blistering plaister is made, resides in a principle called cantharidine. Dissolved in oil, this has been recommended as a substitute for the ordinary *emplastrum cantharidis ;* and M. Bretonneau assures us, that paper imbued with this solution acts with great promptitude and energy. It is said that a tincture prepared with three parts of powdered cantharides and eight of sulphuric ether, is capable of producing vesication in the short space of ten minutes. A blister of this sort is thus made by M. *Trousseau :*—

A piece of blotting-paper is cut of the form and size of the blister that is desired; it is applied upon a sheet of diachylon ; and it imbibes some drops of the extract of cantharides by ether, and it is fixed upon the skin by means of the diachylon.

It appears to us that this is inferior in point of convenience, if not of effect, to the "solution of cantharides," at present much used in London.

It is an omission, perhaps, on the part of Mr. Ure, failing to take notice of it.

The *oil-silk dressing*, says Mr. Ure, may be used with advantage for blistered surfaces. An American writer has suggested the use of finely-carded cotton, which has also been found to answer well.*

"There is an ingenious contrivance for bandaging, which I have seen in Paris. It consists of a light shield, made of thin plated metal, or gum-elastic tissue, to one side of which a band of caoutchouc web is attached, provided at its extremity with a clasp, which fastens to the other side of the shield. These are called, according to the part to which they are adapted, *serre-bras, serre-cuisses*, &c. and are used to contain blisters, issues, &c." 477.

Bloodletting is of course cleverly written by Dr. *Wardrop*, assisted by Mr. Costello. It contains those views and practical directions, which we have already, on previous occasions, had the pleasure of fully noticing. We cannot go over the ground again, but must content ourselves with repeating the favourable opinions we have formerly expressed.

Bone, Diseases of, is a short article by Mr. *Spencer Wells*. He arranges these diseases in the following manner:—

1. Functional affections, or lesions of innervation.
2. Abnormal changes, the result of defect or excess in the nutritive or assimilative powers.
3. Simple and specific inflammation of bone, and its membranes, with the consequence of such inflammation.
4. Morbid growths, deposits, or formations.
5. Malignant diseases.

Speaking of *functional affections of bone*, Mr. Wells believes that their nervous sensibility may be so exalted as to render them incapable of affording support or resistance without pain. He speaks more particularly of the periosteum, and cites two cases where the perichondrium was the seat of this morbid irritability.

While noticing *hypertrophy of bone*, Mr. Wells observes:—it is very rare to find a bone lengthened from hypertrophy; there are, however, in the museum of St. Bartholomew's hospital, sections of two tibiæ so much elongated from this cause, that they have become considerably curved, in order to adapt themselves to the fibulæ, which remain of their natural length. The hypertrophy is confined principally to the cancellated structure.

Bronchotomy is very carefully and completely handled by Mr. *Spencer Wells* also. The practical part of the article received the prize of the Dublin Medico-Chirurgical Society, for 1839. And it seems to us to have deserved it. As it is not long, however, since we devoted some space to Mr. Porter's work, we do not feel ourselves justified in re-entering on the subject at present.

---

* The late Dr. William Young, of Glasgow, was in the habit of applying this material. He assured the editor, that if the blister were removed when rubefaction was produced, and the cotton then applied, the vesication would follow equally well, with but little pain comparatively. In blistering young subjects he always had recourse to this plan.

BUBO is treated by Mr. *Henry James Johnson.* He commences by mentioning the common division of buboes into *specific* and *sympathetic*, and deferring the consideration of the former, limits his observations to the latter.

He gives a slight account of the *superficial* and *deep inguinal glands*—their position, and the quarters from which their lymphatics proceed. He observes that:—" it is not an uncommon circumstance, to find one or more lymphatic glands on the pubes, into which the lymphatics of the prepuce on the dorsum penis enter. The vasa efferentia of these pubic glands, pass, I believe, to the innermost of the inguinal. In inflammatory phymosis, and in acute gonorrhœa, the pubic glands are not unfrequently inflamed; more frequently, if I may trust my own experience, than the inguinal. An indurated absorbent may always be traced to them along the dorsum penis. It occasionally happens, as M. Cruveilhier has remarked, and as practical surgeons must have noticed, that the lymphatics of the penis and the scrotum avoid the glands which are most contiguous, and proceed to the lower ones in the neighbourhood of the saphena vein. A circumstance worth remembering, as it impugns the truth of the current belief, that irritations of the external organs of generation affect only the higher and more internal of the glands of the groin."

Mr. Johnson adds, that the anatomical facts he draws attention to, explain the liability of particular glands to sympathise with particular parts. Mr. Johnson has twice, after tying internal piles, seen smart inflammation of the inguinal glands. In one case, the glands alone were affected; in the other, that of a lady of a highly nervous temperament, the sub-peritoneal cellular tissue appeared to be extensively inflamed, and the irritation seemed to travel by *that* route to the glands in the crural canal.

Boils on the nates, or even a caustic issue in the loins, will give rise to enlargement of the external inguinal glands. In persons who have been subject to femoral hernia, some enlargement of the inguinal glands is not uncommon. The fact is worth mentioning, as inattention to it has led to embarrassment in the operation.

" The inguinal glands enlarge at times, independently of any appreciable irritation in the course of the lymphatics leading to them. In some instances, this would seem to depend on an injury inflicted on them. A person, for example, makes some violent exertion, or takes a long walk, and a bubo follows. Or he has connexion, and without the occurrence of excoriation, or of gonorrhœa, or of any kind of sore, there comes a bubo. No doubt, the greater number of what the French call the *bubons d'emblée,* and of what has been described as ' primary syphilitic bubo,' are no more than simple inflammatory enlargement of a lymphatic gland, occasioned by the mere exertion and excitement of coition." 527.

Mr. Johnson observes, that constitutional states exercise an important influence on both the production and the course of bubo. There are two conditions of the system, the scrofulous and the cachectic, which are more particularly prone to it. For practical purposes, he continues, it might not be amiss to look at bubo under three points of view; as it occurs in a person of a good constitution; or, in one of a scrofulous habit; or, in one of a cachectic—divisions of positive utility, however open to critical objections.

*Bubo in a healthy habit.*—We need not mention the characters of this, but content ourselves with stating that it may end in *resolution, induration, suppuration*, or *sloughing*.

*Suppuration.*—Supposing this to occur, the swelling enlarges, partly from increase of the gland itself, principally from implication of the neighbouring cellular tissue,—a blush of redness appears upon the skin,—the latter becomes adherent to the gland beneath,—slight œdema, from serous effusion in the cellular membrane, precedes "bogginess," from sero-purulent infiltration into it and into the gland—and fluctuation, from concentration of the fluid into one chamber, follows. Mr. Johnson remarks, that it is often hard to say, in the stage of "bogginess," whether there is matter, or whether there is not.

We need not pursue the description of the progress of the abscess to pointing, nor of what occurs when it has given way. Mr. Johnson remarks that:—"Obstinate ulcerations and sinuses are more liable to happen if the patient persists in an injudicious amount of exercise, or in venereal or dietetic excesses. A carpenter had a sinus from bubo, in his right groin; it resisted setons, division, and every thing that I could think of, until he totally discontinued his employment. He had been much in the habit of sawing deal planks, with his right thigh flexed, and his knee pressing on the plank. It is a common enough occurrence for debauchees to continue venereal indulgences with sinuses in the groin, and I have seen these refuse to heal, till continence was submitted to. The gland seldom remains in a morbid state, so as to keep up irritation and ulceration in the parts about it, unless the patient is guilty of great negligence or impropriety. When this is the case, the gland may lie at the bottom of the sinus or beneath the ulcer, surrounded, perhaps, with diseased cellular membrane, itself partially disorganized, and acting like a foreign body."

*Sloughing.*—Usually the patient or the surgeon is to blame if this occurs, at least to any extent. Mr. J. once saw a gland slough out completely. The patient, a young gentleman with a suppurating bubo, *would* go out for a hard day's hunt.

*Bubo in a scrofulous habit.*—Mr. Johnson is disposed to advance the following positions:—

1. Persons of a decidedly scrofulous habit are particularly prone to bubo, and the tendency is to affection of several glands.

2. Bubo, when it occurs, goes through its stages with extreme dilatoriness.

3. Resolution is much less frequent than in the healthy. Induration is very common. Suppuration is common too.

4. When suppuration has occurred, the following condition of the parts is apt to supervene.

"A considerable swelling occupies the groin, sometimes pretty uniform, more frequently irregular upon its surface; the integument is of a bluish, and variegated, or, rather, of a brick-dust red: on pressure, the swelling feels remarkably boggy, elasticity, however, predominating in one part, œdematous pitting in another. Amidst the 'bogginess' and tumefaction, hard lumps may be distinguished, evidently indurated glands: the tenderness is usually inconsiderable, and the seeming amount of disease contrasts strongly with the general absence of pain. If the suppuration has been recent, there are ulcerated apertures, of greater or less size, leading, perhaps, to an exposed gland: if the case is more ancient, those apertures may have closed completely, or, which is more common, they have ended in sinuses, generally leading to a gland altered in its structure.

The discharge, when there is any, is thin, and I have seen it curdy. There is a strong disposition to repeated and partial attacks of suppuration. The duration of this morbid state is always prolonged, and sometimes to a great extent." 528.

At last, however, the swelling subsides, the sinuses close, and the enlargement of the glands may disappear.

*Bubo in a cachectic habit.*—The cachectic condition of system is usually brought on by the injudicious exhibition of mercury, though, in the lower classes, inadequate or improper nutriment and intemperance give birth to it.

The cachectic bubo, says Mr. Johnson, is characterised by the predominance of the suppurative and the ulcerative processes, and by a marked tendency to phagedæna or to sloughing.

Suppuration, when it occurs, is usually extensive; the skin is blue, and disposed to be widely thinned and undermined; the cellular membrane is involved to the same or a greater degree, the absence of the limitations to abscess in a healthy person being marked; the pain is frequently severe, although it is occasionally inconsiderable. Unless an opening be made pretty early, and not unfrequently in spite of every precaution, the skin ulcerates or sloughs freely; a large cavity, with sloughy-looking walls, and with diseased glands, more or less disclosed, is now laid open; granulations rise feebly and irregularly, ulcerating or sloughing, perhaps, after they are formed; the edges of the aperture may continue to ulcerate, and look picked and jagged; or they may ulcerate in one direction and granulate in another; the surrounding cellular membrane still suppurates irregularly or sloughs, so that sinuses open into the old cavity, or fresh ulcerations form in the skin.

A patient in this state is exposed to phagedæna or to gangrene. Any thing which tends to impair his powers may induce the one or the other; and mercury, especially, will do so. But sometimes an irritating application has the same effect. He has seen paring the edges of a cachectic bubo bring on phagedæna, and ulceration of the femoral artery.

The more usual course of cachectic bubo is to lapse into a chronic state. The cavity granulates to a certain extent, its size being proportionally diminished; but the granulations are unhealthy, dark, imperfectly organized, disappearing, or even sloughing readily; the gland is probably still diseased, and often palpably is so; the discharge is thin, copious, and irritating; the edges of the sore, either callous or ulcerating fretfully; the surrounding skin blueish; and the cellular membrane thickened, or traversed by sinuses, or morbid in some other fashion.

But even these cases are curable sooner or later.

*Treatment.*—Confining his attention to the non-specific form of *Bubo*, Mr. Johnson divides it into *general* and *local*. On the former we have not space to dwell.

The *local treatment* will vary as the bubo is in its formative stage, or in that of induration, or of suppuration, or of ulceration. And the *kind* of bubo will modify it also. Leeches, evaporating lotions, or hot fomentations and even poultices are means adapted for the first stage. Mr. Johnson has thought that leeches were of more benefit, when placed around than on the inflamed glands. The leeches may be repeated, if the inflammation continues, or augments, and if they seem to be of service; for sometimes they aggravate the tenderness and swelling, and obviously disagree. In scrofulous and cachectic patients, leeches must be used more sparingly, or even avoided altogether. Supposing that these means fail to induce resolution, the best chance of obviating suppuration is from exciting irritation on the surface. This, Mr. Johnson believes, is best done by a blister, either in its ordinary form, or in that of the liquor lyttæ. He has, on several occasions, seen a bubo apparently *certain* to suppurate, resolved by one or two blisters. The nitrate of silver has been used for the same purpose, by the late Dr. Wallace, of Dublin—and the sulphate of copper, the chloride of zinc, the tincture of iodine, the bichloride of mercury, &c., have been employed with the same view.

*Local Treatment of Indurated Bubo.*—Leeches and cold lotions are generally useless, or worse. Warm applications, more particularly poultices, are occasionally beneficial, and a chronic bubo will now and then subside under incessant poulticing, which has resisted more active measures. Perhaps what are called discutient plaisters, act more by maintaining warmth and moisture than by any more recondite virtues; and the mercurial plaister, the iodine plaister, the emplastrum ammoniaci cum hydrargyro, have all had their patrons, and have all been of service. Stimulating liniments and ointments, such as the ointment of the iodide of potassium, of mercury, of the tartar emetic in a weak form, may be resorted to. And decided irritation of the surface by the tincture of iodine, by the nitrate of silver, by blisters, &c. is frequently very desirable. Mr. Johnson has seen both good and harm from compression. But indurated bubo will sometimes remain long, and inflame and suppurate at last. Perhaps this is a good thing. A blister will sometimes flog up the indolent action, and determine the formation of pus.

*Local Treatment of Suppurating Bubo.*—If resolution is clearly unattainable, it is best to accelerate suppuration. The blister, which, perhaps has been applied with the view of dispersing the enlargement, will generally, if it fails in that, expedite the suppurative process. When this has occurred, the following points present themselves for consideration:—1st. Is it better to make an artificial, or to wait for a natural opening? 2. Is an opening by a cutting instrument, or one by caustic preferable? 3. Shall we practise an early opening or a late, a small or a free one?

1. Generally the abscess should not be allowed to give way spontaneously. But in the scrofulous bubo, where small foci of suppuration are often forming, and the cellular membrane is a good deal implicated, the case often does better without the lancet than with it. The frequent incisions worry the patient, and seem to irritate the skin.

2. Mr. Johnson thinks that there are few cases in which an opening by caustic is justifiable. If a judicious opening relieves all tension, and properly evacuates the matter, the thin blue skin, even of the cachectic bubo (for few would dream of employing caustic in the simple or the scrofulous varieties,) will revive marvellously under generous diet, and stimulants, and tonics, accompanied with appropriate local management.

"I confess that my predilections are rather for early openings. Even in the stage which precedes the perfect formation of pus, and its collection into a single cavity, a moderately free opening has frequently appeared to me to check the progress of the bubo, and materially limit its extent. And several considerations seem to be in favour of this opinion; for, if the bubo depend upon a single gland, it is difficult to understand what objection can lie against an early incision, one that only anticipates what *must* come a little later; and, on the other hand, if several glands are affected, to delay operating would be to offer a premium on a large abscess, a troublesome sore, and a protracted cure, when two or three small incisions might economise both skin and time." 532.

In the scrofulous bubo, we need not be in any hurry to operate.

An early opening almost implies that it is not, or need not be, a large one. Mr. Johnson observes, on the *mode* of making the incision:—" It has appeared to me that something has been gained by attending to the following circumstances:—*First*, to make the puncture in as dependent a part as possible of the general or particular cavity laid open; *secondly*, so to direct the incision that the necessary movement of the limb may not pull the edges asunder; if the abscess be very small, a vertical incision answers best; if larger, it should be oblique, forming an angle of forty degrees, or thereabouts, with the ligament of Poupart.

It not unfrequently happens, that a cavity of some size presents, as it were, two chambers, connected by a more constricted portion. The skin is thin, and an opening is necessary in two places, separated by a greater or less interval from one another. The intermediate part of the cavity does not close with readiness, and it may be requisite at a later period to lay the two openings into one. But I have often gained time, and saved skin, by passing a seton of calico or linen rag, in the first instance, from one opening to the other." Of course the seton tape is only loosely passed, and it may be employed where the patient is anxious to avoid a large scar, or where, from the size of the cavity, we are unwilling to sacrifice skin.

In the cachectic bubo the incisions must often be more free than in the other forms; the cavity being larger, and the skin more undermined. The surgeon should, on this account, take care not to wait too long before he has recourse to the knife.

We need not follow our author into the management of the bubo after the opening, Mr. Johnson remarks on the *local treatment of Sinuses*;—" If these are immediately beneath the skin, and of moderate extent, the simplest plan is to lay them open. But if very long, a seton is preferable; or they may be incised to a convenient extent, and a seton passed through the remainder. If a sinus passes deep, as it sometimes will, it cannot be laid open properly. Injections of a *very* strong solution of the nitrate of silver, or, as Sir Benjamin Brodie, has advised, the introduction of a probe coated with a thin film of it, or a bougie smeared with the nitrate of silver ointment, have all been found to succeed." It is useless, or worse, attempting to heal a sinus leading to a diseased gland or altered cellular tissue.

Speaking of *diseased glands*, which in scrofulous and cachectic buboes, may sometimes be seen in the shape of pale-coloured projections at the bottom of suppurating cavities, or the cavity may be partly formed at their expense, or ,lastly, such a gland may form the termination of a sinus, Mr. Johnson observes, that occasionally the gland is so disorganized that it sloughs away, or may be pulled away, but more frequently it is firmly fixed, and either bits only are separated, or, what is quite as frequent, the gland gradually regains its normal state.

Burns and Scalds are commenced by Mr. *Alexander Ure*. As the article will be continued in the next Part, we shall defer, till that appears, our notice of it.

